# Track Fastener Defect Detection Model Based on Improved YOLOv5s

**DOI:** 10.3390/s23146457

**Published:** 2023-07-17

**Authors:** Xue Li, Quan Wang, Xinwen Yang, Kaiyun Wang, Hongbing Zhang

**Affiliations:** 1 School of Mechanical Engineering, Lanzhou Jiaotong University, Lanzhou 730070, China; 2 Shanghai Key Laboratory of Rail Infrastructure Durability and System Safety, Tongji University, Shanghai 201804, China; 3 State Key Laboratory of Traction Power, Southwest Jiaotong University, Chengdu 610031, China

**Keywords:** track, fastener, defect detection, YOLOv5s, attention mechanism, BiFPN, data enhancement

## Abstract

Defect detection of track fasteners is a prerequisite for safe and reliable railroad operation. The traditional manual visual inspection method has been unable to meet the growing demand for railroad network inspection in China. To achieve the need for accurate, fast, and intelligent detection of rail fasteners, this paper proposes a rail fastener defect detection model based on improved YOLOv5s. Firstly, the convolutional block attention module (CBAM) is added to the Neck network of the YOLOv5s model to enhance the extraction of essential features by the model and suppress the information of minor features. Secondly, a weighted bidirectional feature pyramid network (BiFPN) is introduced to realize the multi-scale feature fusion of the model. Finally, the K-means++ algorithm is used to re-cluster the dataset to obtain the anchor box suitable for the fastener dataset and improve the positioning ability of the model. The experimental results show that the improved model achieves an average mean precision (mAP) of 97.4%, a detection speed of 27.3 FPS, and a model memory occupancy of 15.5 M. Compared with the existing target detection model, the improved model has the advantages of high detection accuracy, fast detection speed, and small model memory occupation, which can provide technical support for edge deployment of rail fastener defect detection.

## 1. Introduction

As of early 2023, the total mileage of China’s railroads in operation has exceeded 155,000 km, and according to China’s medium- and long-term railroad development plan, the total mileage of China’s railroads is expected to reach 175,000 km by 2025 and 200,000 km by 2030 [1]. With the continuous improvement of China’s railroad transportation network, the safety and reliability requirements of railroad operations have are higher. Track fasteners are an essential part of fixed rails. Their role is to maintain the gauge and prevent the longitudinal displacement and lateral displacement of the rail relative to the rail sleeper, and then to protect the safety of train traffic. However, with the increasing running time, speed, and frequency of the train, the fastener system is subject to long-term impact and vibration. As a result, the problem of fastener defects such as bullet fracture, loss, and displacement occurs, seriously affecting the safety of train operation and the line’s reliability. Therefore, the defect detection of track fasteners is crucial to the safety of railroad operations.

China’s fastener defect detection is mainly based on manual visual inspection. This method is not only time-consuming and labor-intensive but also inefficient in terms of detection, as detection accuracy is easily affected by subjective factors. To improve inspection efficiency, researchers at home and abroad have developed NDT methods, including vibration signal-based detection [2,3], ultrasonic detection [4], laser detection [5], and machine vision detection [6,7]. In recent years, with the rapid development of artificial intelligence, detection methods based on machine vision, in particular, have received more and more attention and application in the field of track defect detection. In general, machine vision-based detection methods are mainly divided into traditional image processing detection methods and deep learning-based detection methods.

Traditional image processing detection methods require human design and extraction of fastener features, followed by defect recognition classification with trained classifiers [8]. This is largely limited by the subjective feature design and extraction and has limited feature extraction capability for images, so the detection accuracy and detection speed are relatively low. Moreover, it is easily disturbed by factors such as lighting conditions, image noise, and tracking environment in actual detection and needs better generalization ability and robustness. Liu et al. [9] extracted the fastener pyramidal gradient histogram and image local macro texture features and then used a support vector machine (SVM) to identify and classify the fastener defects. Ma et al. [10] extracted the edge features of fasteners using a median filter, improved the Canny edge detection method, and matched the features of defective fasteners using feature templates to achieve real-time detection of fasteners. Gibert et al. [11,12] proposed a fastener detection algorithm based on a multi-task learning framework combined with multiple detectors using a histogram of oriented gradient (HOG) of images to extract fastener features and SVM classifier to classify and identify damaged and missing fasteners. Ou et al. [13] developed a Bayesian hierarchical model of fastener features and structural labels and used an SVM classifier to classify defective fasteners. To solve the problem of a lack of defective fastener samples, Dai et al. [14] proposed a method for generating images of fractured fasteners, and after obtaining a large number of virtual samples of fractured fasteners, drawing on the calculation method of HOG features to extract the slug gradient features in the images and to use principal component analysis for feature dimensionality reduction. Finally, a trained SVM classifier achieved the detection of defective fasteners. Although the above traditional image processing detection methods have improved the detection accuracy to a certain extent, their complex image processing and feature extraction process still cannot improve the detection efficiency of fasteners.

Deep learning-based detection methods mainly use convolutional neural networks (CNN) to learn feature information of detection targets from a large amount of image data for fast and accurate recognition of targets. Compared with traditional image processing methods, it avoids the disadvantage of designing feature information by hand and thus has good generalization and robustness. There are two main types of deep learning-based target detection algorithms: two-stage and one-stage. Two-stage refers to algorithms that require two steps for target recognition based on target candidate frames, mainly R-CNN [15], Fast-R-CNN [16], and Faster-R-CNN [17]. One study [18] uses Faster-R-CNN to detect rail fasteners. Although the detection accuracy is improved, the number of model parameters is large, and the detection speed could be more satisfactory. One-stage refers to the location and class of the target that can be predicted directly without generating candidate regions, mainly the SSD [19] and YOLO [20,21,22,23] series. Compared with other target detection algorithms, the YOLO series algorithm proposed by Redmon et al. has significant advantages in terms of detection speed and accuracy. Hence, it is also widely used in track fastener defect detection. Qi et al. [24] proposed an improved MYOLOv3-Tiny network based on YOLOv3 to achieve higher detection accuracy and a significant improvement in the detection speed of this model compared to Faster-R-CNN. Guo et al. [25] proposed a real-time detection model for tracking fasteners based on improved YOLOv4, and the experimental results showed that the model could achieve 94.4% mAP for fastener detection. Subsequently, Jocher et al. proposed YOLOv5 [26], which is an improvement on YOLOv4 [23] and employs deep learning techniques such as deep separable convolution [27] and fine-grained feature pyramid networks [28] to further improve the detection accuracy and speed of the model. Although the above methods can accurately detect fastener defects, they could be more effective in detecting displaced, lost, and broken fasteners. The poor generalization and robustness of the model make it difficult to apply to engineering practice.

The current fastener defect detection process has problems such as low detection accuracy, slow detection speed, and poor model robustness. This paper proposes a rail fastener defect detection model based on improved YOLOv5s. The main contributions of this paper are as follows:

1. We collected 750 RGB images of the fasteners. The fastener images were augmented using deep convolutional generative adversarial network (DCGAN) and various data enhancement methods. Finally, the images were annotated using LabelImg to build a fastener dataset with a sample size of 3200.
2. CBAM module and BiFPN module are separately introduced in the Neck network of the YOLOv5s model. The feature learning capability of the model is enhanced to achieve multi-scale feature fusion, which further improves the detection accuracy of the model.
3. The K-means++ algorithm re-clusters the anchor boxes, improving the prediction frame’s precision and enhancing the model’s localization ability.
4. The detection performance and robustness of the model are verified using fastener images collected under different environmental conditions.

## 2. Methods and Improvements

### 2.1. YOLOv5 Algorithm

**Figure 1:**
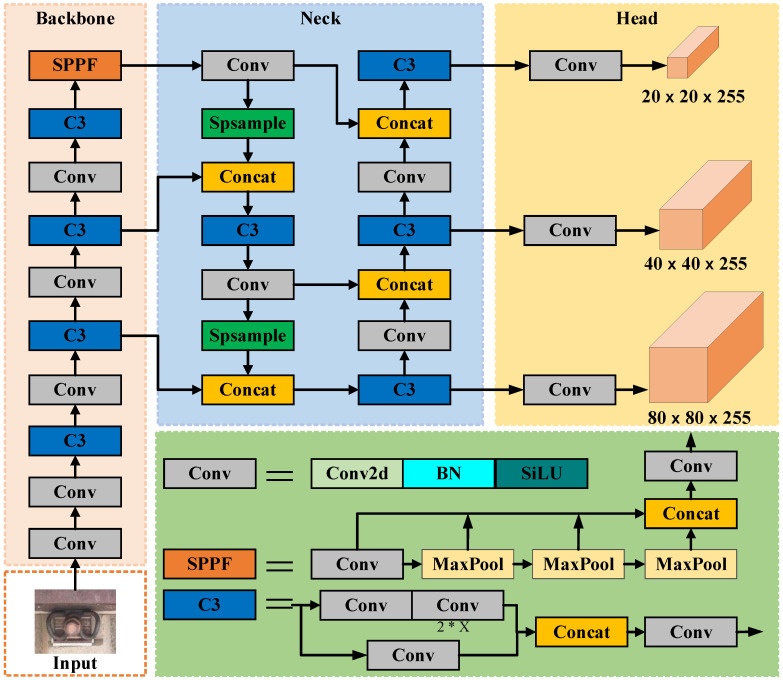
Original YOLOv5 network structure.

The YOLOv5 algorithm is now widely used in various visual inspection situations in rail transportation due to its robust performance in target detection. Its network structure is shown in Figure 1, which mainly consists of four parts: Input, Backbone, Neck, and Head. The Input performs image pre-processing, such as adaptive image scaling, batch normalization, and Mosaic data enhancement on the input image. The Backbone mainly uses CSPDarknet as the backbone network, extracts the features of the image through convolutional layers, performs feature stacking using the C3 module after each convolution operation, and adds the SPPF module to pool the features at the end of the Backbone network to output three feature layers at different scales finally. The Neck network mainly performs feature fusion to enable feature fusion of semantic information from the deep feature layer and location information from the shallow feature layer to enhance the feature learning capability of the model for detection targets. The Head network mainly divides the feature images fused by the Neck network into three different sets of grid scales, large and small, and then uses different anchor boxes to detect the target information in the grid and finally performs decoding detection in the generated actual frames.

**Figure 2:**
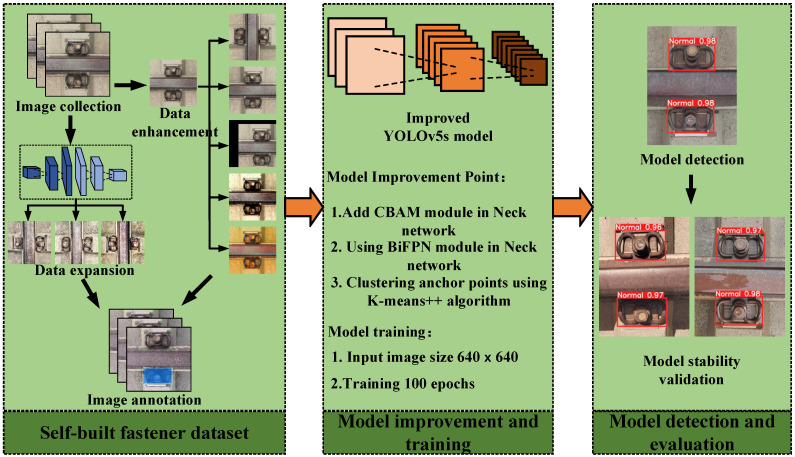
Flow chart of this paper’s research.

The Yolov5 algorithm provides five versions of YOLOv5n, YOLOv5s, YOLOv5m, YOLOv5l, and YOLOv5x depending on the depth and width of the model for different detection needs. To meet the needs of this research, YOLOv5s is selected as the base model of the rail fastener defect detection algorithm, taking into account the requirements of detection precision and detection speed. The specific workflow of this paper is shown in Figure 2.

### 2.2. YOLOv5s Improvement

#### 2.2.1. Attentional Mechanisms

Numerous experiments have demonstrated that the attention mechanism enables the model to focus more on the target region and thus extract more essential features. Currently, the more common attention modules are SE [29], CA [30], and CBAM [31], all of which can improve the detection performance of the model to varying degrees. To enhance the detection performance of the model on the fastener dataset, this paper introduces the CBAM module into the Neck network of the YOLOv5s model. The attention of the model to local and spatial information on space and channels is enhanced, thus reducing the proportion of weights occupied by irrelevant features.

**Figure 3:**
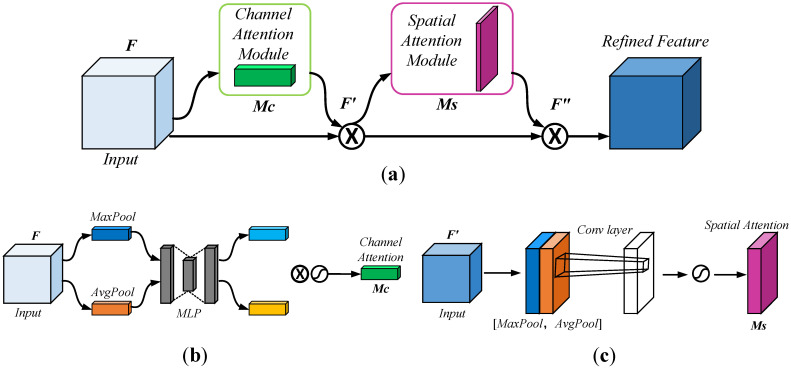
(**a**) Schematic of the overall structure of CBAM; (**b**) schematic of the channel attention module; (**c**) schematic of the spatial attention module.

CBAM mainly comprises a channel attention module and a spatial attention module, and its structure is shown in Figure 3a. When CBAM receives the feature maps in the network, it processes them sequentially along two dimensions, channel and space. Then, it multiplies the processed feature maps with the input ones to achieve adaptive feature optimization.

The specific role of CBAM in convolutional networks is that, given an input feature map F, the feature map $F^{\prime }$ is first computed by mapping the weight coefficients $M_{C}$ of the channel attention mechanism. Then, the final feature map $F^{\prime \prime }$ is calculated by mapping the weight coefficients $M_{S}$ of the spatial attention mechanism. The computation process is as follows:

(1) $$F^{\prime }=M_{C}(F)⊗F$$

(2) $$F^{\prime \prime }=M_{S}(F^{\prime })⊗F^{\prime }$$

In the channel attention module, as shown in Figure 3b, the input feature map F is mapped by average pooling and maximum pooling and then processed by the shared multi-layer perceptron (MLP) mapping to obtain two features and sum them. Then, the weight coefficients of the Sigmoid activation function obtain $M_{C}$. Finally, the obtained weight coefficients $M_{C}$ are multiplied with the input original feature map F to generate the scaled new feature map $F^{\prime }$. The $M_{C}$ is calculated as follows:

(3) $$M_{C}=\sigma (MLP(AvgPool(F))+MLP(MaxPool(F)))$$

where σ denotes the Sigmoid activation function, MLP is the multilayer perceptron, AvgPool is the average pooling operation, and MaxPool is the maximum pooling operation.

In the spatial attention module, as shown in Figure 3c, the feature map $F^{\prime }$ generated by the channel attention module is average pooling and maximum pooling operations to obtain two channel maps. The resulting channel mapping of these two backgrounds is subjected to Concat operation. Then, a 7 × 7 convolution operation is reduced to one channel, and a Sigmoid activation function is used to obtain the weight coefficient $M_{S}$. Finally, the new feature map is obtained by multiplying the weight coefficient $M_{S}$ with the feature map $F^{\prime }$. The $M_{S}$ is calculated as follows:

(4) $$M_{S}=f^{7\times 7}[(AvgPool(F^{\prime });MaxPool(F^{\prime }))]$$

where $F^{\prime }$ denotes the feature map generated by the channel attention mechanism, and $f^{7\times 7}$ represents the 7 × 7 convolution operation.

**Figure 4:**
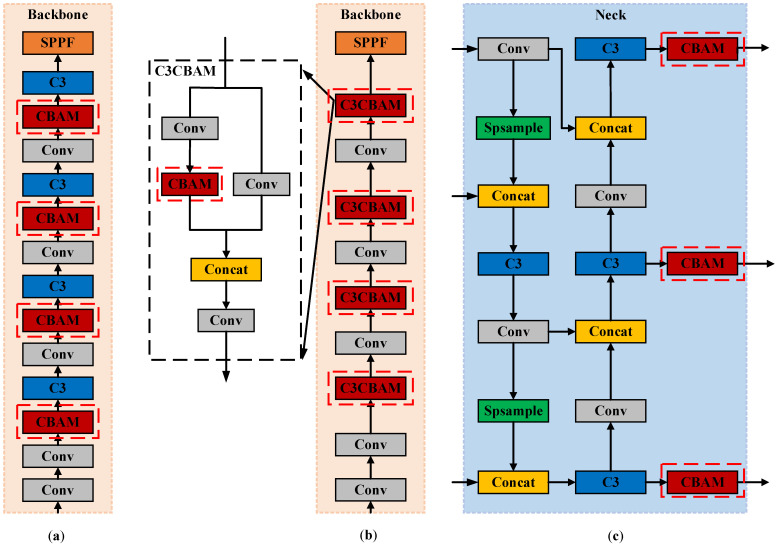
CBAM modules are added: (**a**) after each convolution operation of Backbone; (**b**) in each C3 module of Backbone; (**c**) after the C3 module of Neck network.

In this paper, we are adding SE, CA, and CBAM attention modules separately to the YOLOv5s model Neck network, and adding the attention models to different positions of the YOLOv5s model, as shown in Figure 4. Through experimental verification, adding the CBAM attention module in the Neck network has the most noticeable performance improvement effect on the model.

#### 2.2.2. BiFPN Structure

In the fastener dataset, there are different image resolutions and slight variability in the size of the labeled boxes for each inspection category. During network training, as the training depth of the network increases, the position information of the detected target becomes weaker, resulting in a larger position loss of the target. To solve this problem, this paper proposes to introduce the BiFPN module in the Neck network of the YOLOv5s model to improve the detection precision of the model.

**Figure 5:**
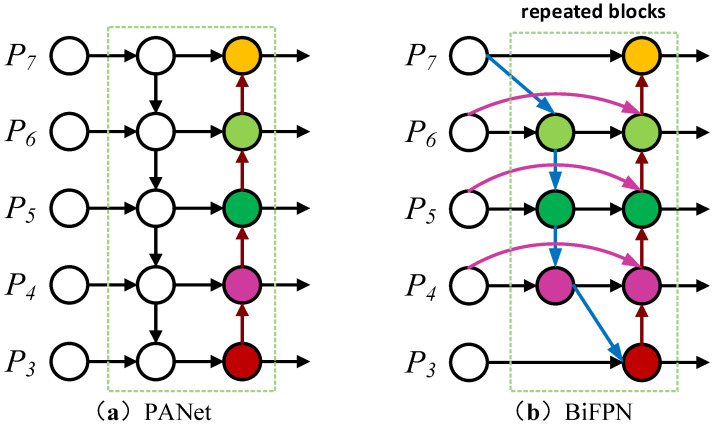
PANet structure and BiFPN structure.

A simple bidirectional fused feature pyramid PANet [32] structure is used in the Neck of the original YOLOv5s network. The network structure mainly achieves feature fusion of shallow location information and deep semantic information through bottom-to-top and top-to-bottom bi-directional feature fusion while shortening the information path between the bottom and top layers. However, the PANet structure has two nodes with only one side input but no feature fusion, which is of little use for feature fusion and adds extra parameters and computation. The BiFPN structure is improved on the original PANet structure by removing two redundant nodes at the top and bottom of the PANet structure and increasing the degree of fusion of different features by adding further weights to the input features to enhance the multi-scale feature fusion of the model. The structures of PANet and BiFPN are shown in Figure 5.

Since the input feature maps have different resolutions, the BiFPN structure assigns weights to the additional feature layers according to their contribution to the network when performing feature fusion. The model training focuses on learning features with larger-weight assignments and performs multi-scale feature fusion iteratively. The BiFPN weighting formula is

(5) $$O=\sum_{i}\;\frac{w_{i}}{e+\sum_{j}w_{j}}I_{i}$$

where O is the output value of the node, $I_{i}$ is the input value of node i, $w_{i}$ is the weight corresponding to the input of node i, j is the total number of input nodes, and *e* is a minimal learning rate with stable values usually of e=0.0001. The following is an example of feature fusion on node $P_{6}$, which is calculated as follows:

(6) $$P_{6}^{td}=Conv(\frac{w_{1}P_{6}^{in}+w_{2}Resize(P_{7}^{in})}{w_{1}+w_{2}+e})$$

(7) $$P_{6}^{out}=Conv(\frac{w_{1}^{\prime }P_{6}^{in}+w_{2}^{\prime }P_{6}^{td}+w_{3}^{\prime }Resize(P_{5}^{out})}{w_{1}^{\prime }+w_{2}^{\prime }+w_{3}^{\prime }+e})$$

where $P_{6}^{td}$ is the intermediate features generated in the top-down feature fusion process, $P_{6}^{out}$ is the output features developed in the bottom-up feature fusion process, $P^{in}$ is the down-sampling operation on the input features, Resize is the up-sampling function, w is the learned parameter, $P^{out}$ is the output features after fusion, and Conv is the convolution operation function.

#### 2.2.3. Anchor Box Optimization

The original YOLOv5s model for detecting track fasteners has problems such as inaccurate target positioning, which is caused by the poor positioning of fasteners due to the use of YOLOv5s preset anchor boxes. The preset anchor boxes in the YOLOv5s network are obtained based on the clustering of the COCO [33] dataset. The dataset is mainly based on familiar objects with more categories. Hence, the anchor boxes obtained by clustering are better for detecting everyday objects. However, there is a significant positioning error in the detection of track fasteners, which affects the detection accuracy of the model.

The anchor box parameters that are more suitable for the fastener dataset are obtained to improve the model’s accuracy for detecting defective fasteners. In this paper, the fastener dataset was first clustered using the K-means clustering algorithm to obtain new anchor boxes. The obtained anchor boxes were then used to train and test the fastener dataset, and it was found that there was little improvement in the detection accuracy of the model. The reason for the analysis is that the initial clustering centroids of the K-means algorithm are randomly selected, and the size of each category of fastener labeling box in the fastener dataset does not change much, which leads to a more significant impact on the division of clusters.

To solve the problem of random selection of cluster centroids by the K-means algorithm, this paper uses the K-means++ algorithm to solve this problem. The K-means++ algorithm is optimized for the selection of initial clustering centroids. This is performed to increase the distance of the k initial clustering centroids as much as possible, effectively improving the clustering efficiency. The specific steps are as follows.

Step 1: Randomly select one from the input set N as the first clustering center O1;

Step 2: Calculate the distance D(x) from each sample point *x* to the nearest existing cluster centroid and also calculate the probability P(x) of each sample being identified as the next cluster center as

(8) $$P(x)=\frac{D{\left(x\right)}^{2}}{\sum_{x\in N}D{\left(x\right)}^{2}}$$

(9) D(x)=1−IoU

where *IoU* denotes the intersection ratio of the clustering center to the labeled box.

Step 3: Repeat step 2 and select the sample with the most significant P(x) as the next cluster center until k cluster centroids are chosen;

Step 4: By calculating the IoU values of each sample frame to the k cluster centroids and assigning them to the cluster with the most significant value IoU;

Step 5: Calculation of new clustering centers based on the resulting categories;

Step 6: Repeat steps 4 and 5 until the size of the clustering centroids no longer changes, and finally, obtain the new anchor parameters.

Through the experiments, it was found that the anchor boxes obtained by clustering with the K-means++ algorithm have the most significant improvement in the detection accuracy of the model. The parameters of the anchor boxes obtained by clustering each algorithm are shown in Table 1.

#### 2.2.4. Improving the Model Structure

**Figure 6:**
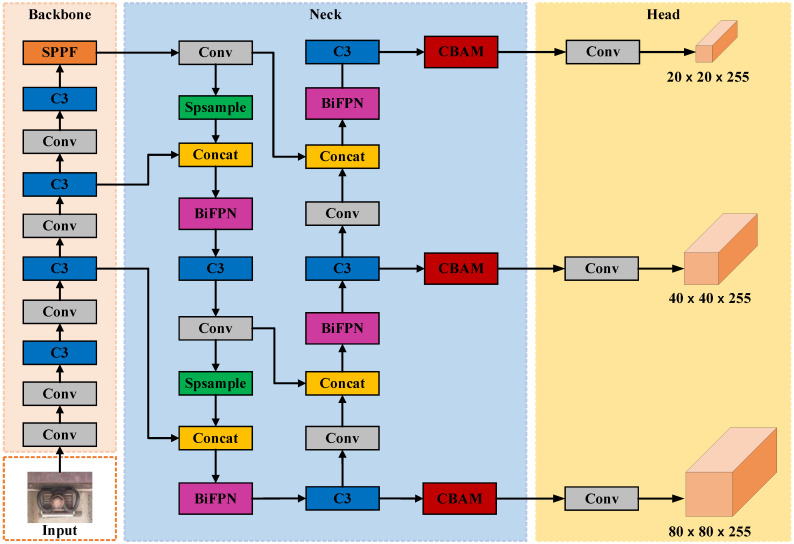
Improved YOLOv5s network structure.

The experimental results finally verify the CBAM module in this paper. It is determined that the CBAM module is added to the YOLOv5s model Neck network, and the location of the addition is shown in Figure 4c. The introduced BiFPN structure in the Neck network has the most stable and noticeable performance improvement for the model in fastener defect detection. The improved model structure is shown in Figure 6.

## 3. Fastener Dataset

### 3.1. Image Collection

Most of the commonly used rail fastener datasets currently use grayscale images taken by onboard CCD cameras, which lose the color features of the images. In addition, the long distance of the camera makes the image clarity low and of poor quality, which leads to insufficient learning of the features of the fasteners by the neural network, thus affecting the detection performance of the model. To solve this problem, this paper uses the camera to shoot vertically with a height of 800 mm from the fastener and obtains RGB color images of the fastener with an image resolution size of 1200 × 900. After several acquisitions to remove the distorted and blurred images, 750 images of fasteners were finally obtained.

### 3.2. Data Expansion

In the actual acquisition process of fastener images, collecting many defective ones is difficult because the inspection and maintenance of railroad line fasteners are very timely. Therefore, the problem of imbalance between positive and negative samples in the fastener dataset arises, eventually leading to the detection precision of defective fasteners being much less than that of standard fasteners. To solve this problem, this paper proposes to use a deep convolutional generative adversarial network (DCGAN) to generate many images of defective fasteners to solve the positive and negative sample imbalance problem.

**Figure 7:**
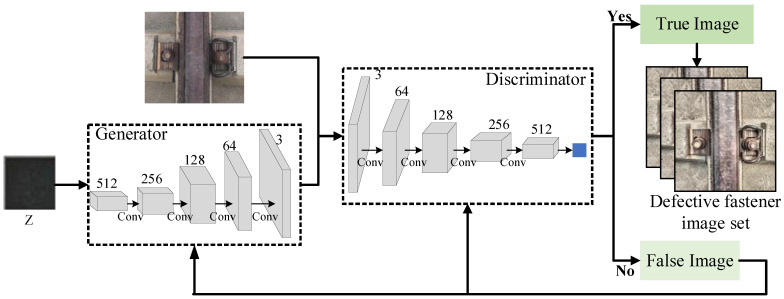
Structure of the DCGAN network.

The structure of the DCGAN network is shown in Figure 7, which mainly consists of a generator and a discriminator. The generator inputs a random noise, Z, and outputs a sample of the generated image. The discriminator receives the generated image samples from the generator and the actual image samples from the dataset and discriminates and distinguishes the real examples from the generated ones. Unsupervised learning is performed by playing two neural networks against each other until the model cannot determine the authenticity of the input samples, and the final output generates image samples.

**Figure 8:**
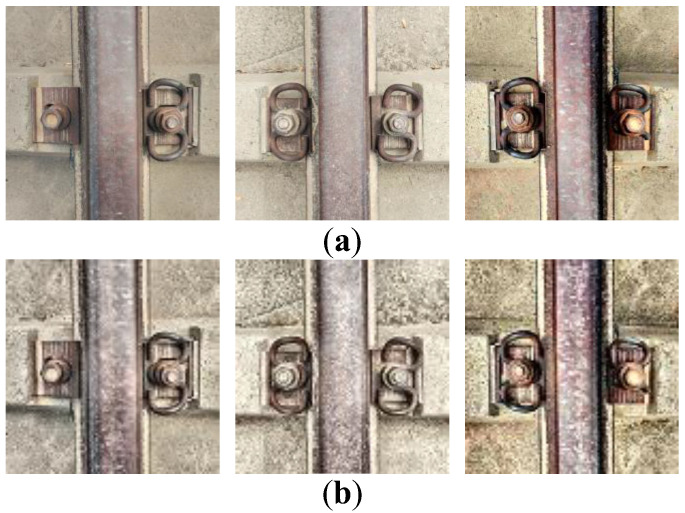
Expanded data samples using DCGAN: (**a**) original data; (**b**) expanded data.

The DCGAN network is used to expand the defective fastener images, and the comparison effect of imperfect fastener images before and after the expansion is shown in Figure 8.

### 3.3. Data Enhancement

**Figure 9:**
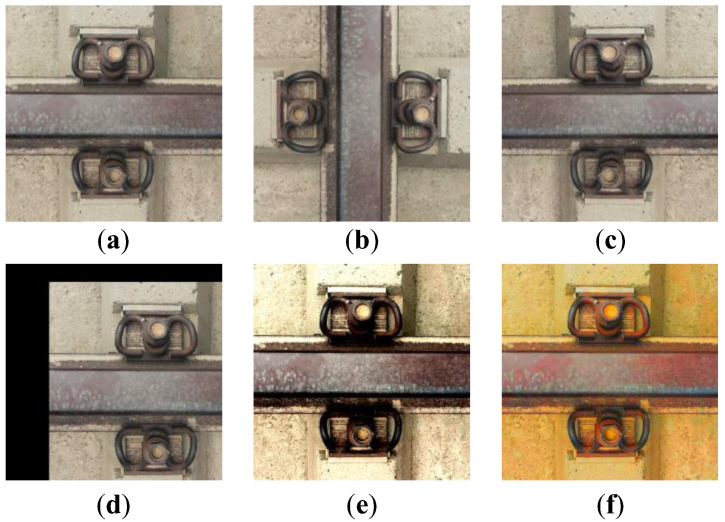
Data enhancement: (**a**) original image; (**b**) rotate; (**c**) horizontal flip; (**d**) translate; (**e**) contrast enhancement; (**f**) color enhancement.

Different weather and environmental factors cause differences in the imaging of fastener images, making the model detection performance unstable. Therefore, data enhancement [34] methods such as image flipping, panning, contrast enhancement, and color enhancement were used to simulate the fastener images under different conditions. Data enhancement can be used to expand the dataset further, extend the richness of the fastener image set, and prevent overfitting due to the small number of samples in the dataset. The data augmentation methods are shown in Figure 9.

### 3.4. Image Annotation

**Figure 10:**
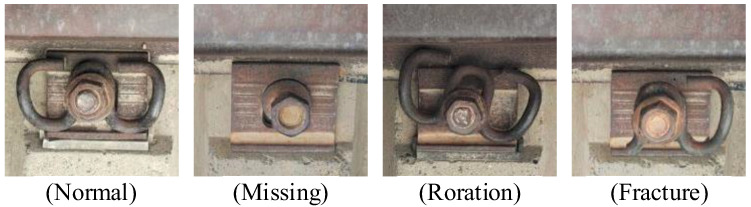
Fastener labeling category.

The final 3200 fastener images were obtained by data enhancement. Then, the images were manually labeled using LabelImg [35]. The labeling categories are shown in Figure 10, which are divided into four categories: Normal, Missing, Roration, and Fracture. The labeled files are saved in PASCAL VOC [36] format, and the xml file containing the category and location information is automatically generated after the images are marked. Moreover, the annotated dataset is divided into the training set, test set, and validation set in the ratio of 8:1:1. The number of samples in the training set is 2560, the number of samples in the test set is 320, and the number of pieces in the validation set is 320.

## 4. Experiments and Results

### 4.1. Experimental Environment and Parameter Setting

The experimental environment was configured with Windows 10 as the operating system, NVIDIA Tesla T4 as the GPU model with 16 G of video memory, Python 3.8 as the compilation language, Pytorch 1.7.0 as the deep learning framework, and CUDA 11.0 as the CUDA version. The training parameters were set as follows: the initial learning rate is 0.01, the momentum parameter is 0.937, the weight decay factor is 0.0005, the input image size is 640 × 640, and the Batch_Size is 16. A total of 100 epochs were trained by stochastic gradient descent (SGD) for the whole training process.

### 4.2. Evaluation Indicators

In this paper, the performance of the improved algorithm was evaluated in three respects, detection accuracy, detection speed, and model size, using the evaluation methods commonly used for target detection algorithms.

Detection accuracy evaluation metrics comprise precision, recall, and mean average precision (mAP).

Precision, denoted by *P*, is the probability that the model detects correctly in a single category. It is defined as follows:

(10) $$P=\frac{TP}{TP+FP}$$

Recall, denoted by *R*, is the probability that the model correctly identifies a positive sample in a single category. It is defined as follows:

(11) $$R=\frac{TP}{TP+FN}$$

where TP denotes the number of positive samples detected correctly, FP denotes the number of positive samples detected incorrectly, and FN denotes the number of negative samples detected incorrectly.

The mean average precision (*mAP*) is the area enclosed by the precision and recall curves. It is an overall network performance evaluation metric considering *P* and *R* [37]. Therefore, *mAP* is a more authoritative metric in model performance evaluation, and a larger *mAP* value represents higher detection precision [38]. All the above metrics are calculated when *IoU* = 0.5, *IoU* is the overlap between the generated prediction frame and the rear frame; that is, the ratio between the intersection of the prediction frame and the actual frame and the concurrent set is 0.5. The following formula calculates each of the above metrics:

(12) $$AP=\int_{0}^{1}P\cdot RdR$$

(13) $$mAP=\frac{1}{N}\sum_{i=1}^{N}AP_{i}$$

(14) $$IoU=\frac{A\cap B}{A\cup B}$$

where N is the number of categories in the dataset, A is the predicted frame area, and B is the actual frame area.

The detection speed is evaluated in terms of frames per second (FPS), the number of frames per second that the model processes the image.

The evaluation metrics for model size include GFLOPs and model weight file size. The model weight file size is the model saved after the final training, denoted by Weight.

### 4.3. Attention Mechanism Validation Experiments

These experiments are designed to confirm that adding an attention mechanism to the YOLOv5s model can effectively improve the model’s performance. The following experiments use YOLOv5s preset anchor boxes, using P, R, and mAP as evaluation metrics. The primary investigation aims to investigate the effect of adding different attention mechanism modules to the YOLOv5s model and the impact of adding the attention mechanism modules to varying positions on the model performance.

#### 4.3.1. Adding Different Attention Mechanisms

To investigate the effect of different attention mechanisms on model performance, in this experiment, SE, CA, and CBAM attention mechanism modules were individually added to the YOLOv5s model Neck network. The CBAM module was added, as shown in Figure 4c. Four sets of experiments were designed under the same training and testing parameters. The first group without any added attention mechanism, i.e., the original YOLOv5s model, was the control group, named YOLOv5s. The second group added SE, named YOLOv5s_NeckSE. The third group added CA, named YOLOv5s_NeckCA. The fourth group added CBAM, named YOLOv5s_NeckCBAM. The experimental results are shown in Table 2.

The experimental results in Table 2 show different effects on the detection accuracy of the model after adding three other attention mechanisms. In particular, the detection accuracy of the model improved significantly with the addition of CBAM, and the P and mAP were enhanced by 1% and 1.1%, respectively, compared with the original model of YOLOv5s, and the GFLOPs and FPSs of the three experimental groups were the same. Therefore, adding CBAM is more helpful for accurately detecting fastener targets.

#### 4.3.2. Adding CBAM in Different Locations

To investigate the effect of CBAM addition location on model performance, the same training and testing parameters as described in Section 4.3.1 were used to design four sets of experiments. The first group without any added attention mechanism, i.e., the original YOLOv5s model, was the control group, named YOLOv5s. The second group of experiments added CBAM after each convolution operation in the YOLOv5s model Backbone, as shown in Figure 4a, named YOLOv5s_ConvCBAM. The third group of experiments added CBAM to each C3 of the YOLOv5s model Backbone, as shown in Figure 4b, named YOLOv5s_C3CBAM. The fourth group of experiments added CBAM after C3 of the YOLOv5s model Neck network, as shown in Figure 4c, named YOLOv5s_NeckCBAM. The experimental results are shown in Table 3.

The experimental results in Table 3 show that adding CBAM to C3 of Backbone reduces the GFLOPs of the model, but at the same time, it also reduces the model’s detection accuracy. Adding CBAM after the convolutional layer of the Backbone and adding CBAM to the Neck network can improve the detection accuracy of the model. However, it can be seen that adding CBAM to the Neck network has the most significant improvement in the detection accuracy of the model. Therefore, the CBAM module is more suitable to be added to the YOLOv5s model Neck network.

In summary, according to the experimental results in Section 4.3.1 and Section 4.3.2, we can establish that adding CBAM to the YOLOv5s model Neck network can effectively improve the detection accuracy of the model.

### 4.4. Anchor Box Optimization Experiments

To verify the effect of anchor boxes on the detection accuracy of the model, the models were experimented with using YOLOv5s preset anchor boxes, anchor boxes obtained by clustering with the K-means algorithm, and anchor boxes obtained by clustering with the K-means++ algorithm. P, R, and mAP were used as evaluation metrics, and they were trained and tested under the same hardware and software environment and training parameters. The testing results are shown in Table 4.

The experimental results show that the detection accuracy of the model with the K-means++ algorithm is significantly higher than that of the YOLOv5 preset anchor box model and the model using the K-means algorithm. Compared to the YOLOv5 pre-built anchor box model, the P and mAP of the model using the K-means++ algorithm improved by 1.1% and 1.4%, respectively. Therefore, it shows that the anchor boxes obtained by clustering using the K-means++ algorithm are more suitable as anchor boxes for the fastener dataset.

### 4.5. Performance Analysis of the Improved Model

**Figure 11:**
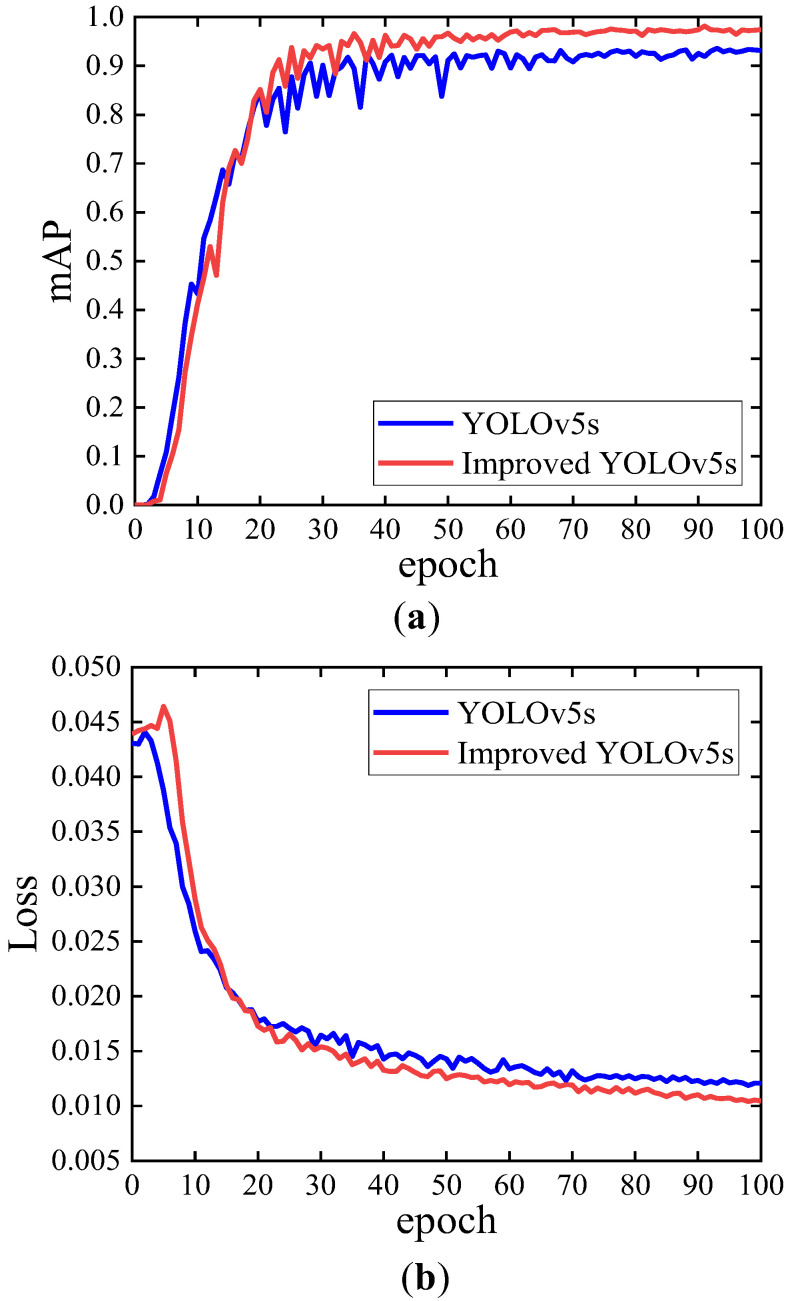
Model performance curves before and after improvement: (**a**) mAP change curve; (**b**) loss change curve.

Figure 11 shows the comparison of mAP curves and loss curves of the improved YOLOv5s model and YOLOv5s model in this paper. The mAP curves in Figure 11a show that the YOLOv5s model mAP values are higher than those of the improved YOLOv5s model in the first 20 epochs. After the 20th epoch, the mAP value of the improved YOLOv5s model exceeds the mAP value of the YOLOv5s model. The mAP values of the improved YOLOv5s model stabilized after the 50th epoch, while the YOLOv5s model stabilized only after 70 epochs. As seen from the loss curves in Figure 11b, the loss value of the YOLOv5s model decreases faster than that of the improved YOLOv5s model in the first 20 epochs. After the 20th epoch, the loss value of the improved YOLOv5s model is significantly lower than that of the YOLOv5s model. The convergence value of the loss curve of the improved YOLOv5s model is lower and converges to 0.01.

**Figure 12:**
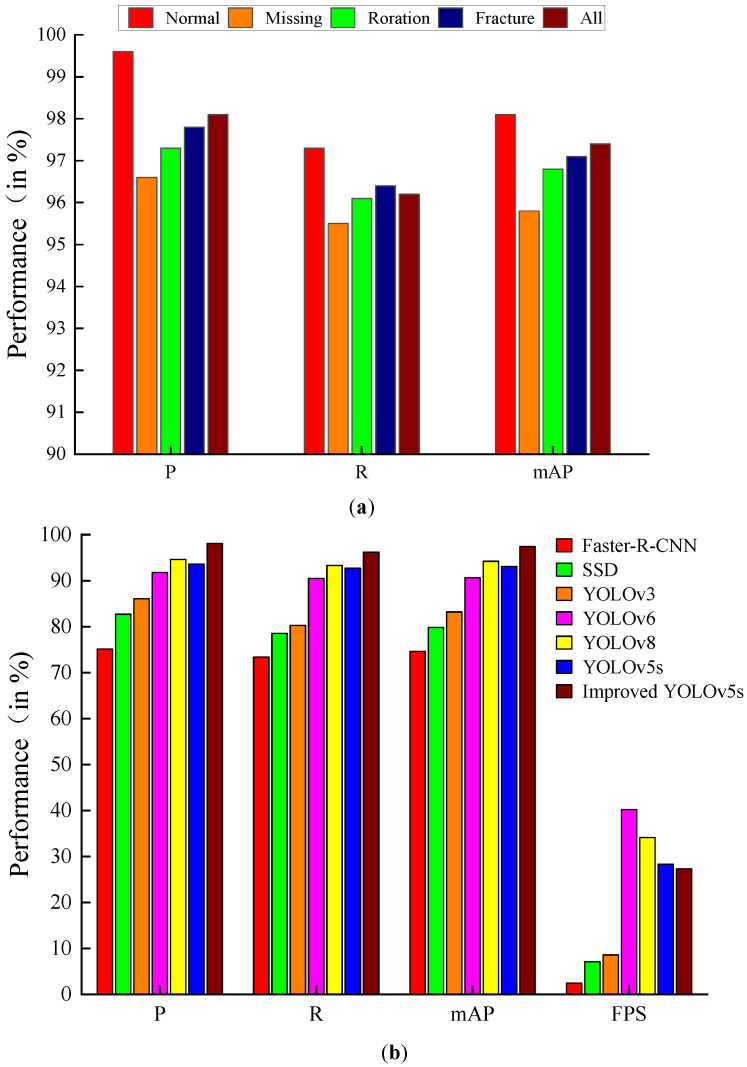
(**a**) Comparison bar graphs of detection precision for different label classes; (**b**) comparison bar graphs of detection precision and detection speed for different detection algorithms.

Figure 12a shows the comparison of the detection accuracy of the improved model in this paper for different label classes. The bar chart shows that the improved model achieved 98.1%, 96.2%, and 97.4% for P, R, and mAP, respectively, for all classes detected. It can also be seen that the P, R, and mAP detected by the label class Normal are significantly higher than the other three classes and higher than the values of P, R, and mAP for all classes. Although the mAP of label class Missing, Roration, and Fracture is smaller than that of fasteners with label class Normal, the mAPs of Missing, Roration, and Fracture reach more than 95%, respectively, which fully meets the detection needs of defective fasteners.

### 4.6. Ablation Experiments

We conducted ablation experiments for the three improvements proposed in this paper. A randomized combination of three improvements using the K-means++ algorithm to re-cluster the new Anchor, adding a CBAM attention mechanism to the Neck network, and using a BiFPN network structure in the Neck network is used to verify the impact of each improvement on the model performance. The experiments were divided into eight groups and trained and tested on the self-built fastener dataset using the same software, hardware environment, and training parameters. The experimental results are shown in Table 5.

Experimental results show that among the single improvements, the new Anchor has the most significant advancement in model detection accuracy relative to the original YOLOv5s model compared to the other two improvements. P and mAP were improved by 1.1% and 1.4%, respectively, over the original YOLOv5s model and did not affect the FPS and GFLOPs of the model. Therefore, the new Anchor using the K-means++ algorithm for re-clustering is more suitable for defect detection of fasteners. In the two–two combination improvement, using the new Anchor and then adding CBAM and BiFPN separately is more significant than using YOLOv5s to preset the anchor frame and then adding CBAM and BiFPN separately to improve the model detection accuracy, with P and mAP improving by 0.9%, 0.8% and 1.3%, 1.2%, respectively. It can be seen that the model is best improved after using the re-clustered Anchor. By adding all three improvements to the original model at the same time, the detection accuracy of the model has been substantially enhanced. Compared with YOLOv5s, original model P and mAP improved by 4.5% and 4.3%, respectively, and GFLOPs only increased by 7.0%, although the FPS of the model slightly decreased while still meeting the detection requirements.

### 4.7. Comparison Experiments

To further validate the detection performance of the improved model, the model in this paper was compared with the existing mainstream target detection algorithms Faster-R-CNN, SSD, YOLOv3, YOLOv6 [39], YOLOv8 [40], and YOLOv5s for experiments. Experiments were conducted using a self-built snapshot dataset with the same hardware and software environment and the same training and testing parameters. A comprehensive comparison of model performance was performed using P, R, mAP, weight file size (Weight), GFLOPs, and FPS. The experimental results are shown in Table 6.

This can be seen based on the data in Table 6 and the bar chart in Figure 12b. In terms of detection accuracy, the mAP of the improved model reached 97.4%, which is a 4.3% improvement compared to the original model of YOLOv5s. Compared with Faster-R-CNN, SSD, YOLOv3, YOLOv6, and YOLOv8, the mAP of the improved model improved by 22.8%, 17.6%, 14.2%, 6.8%, and 3.2%, respectively. The detection speed of the improved model is 27.3 FPS. 24.8 FPS, 20.2 FPS, and 18.7 FPS, which is improved compared with Faster-R-CNN, SSD, and YOLOv3. Although the detection speed is less than YOLOv6, YOLOv8, and YOLOv5s, it still meets the fastener detection requirements. In terms of model size, the Weight and GFLOPs of the improved model are much smaller than those of Faster-R-CNN, SSD, YOLOv3, YOLOv6, and YOLOv8. Although the Weight and GFLOPs of the improved model are slightly larger than those of the original YOLOv5s model, the GFLOPs increase by 7.0% compared to the original YOLOv5s model. The mAP of the improved model increased by 4.3%. In summary, compared with other detection target detection models, the improved model in this paper has certain advantages in detection accuracy, detection speed, and model scale and performs better on the fastener dataset.

### 4.8. Algorithm Visualization and Its Stability Verification

The fastener inspection process will inevitably encounter different weather conditions, and different weather will lead to differences in the imaging of images, ultimately resulting in unstable model detection accuracy.

**Figure 13:**
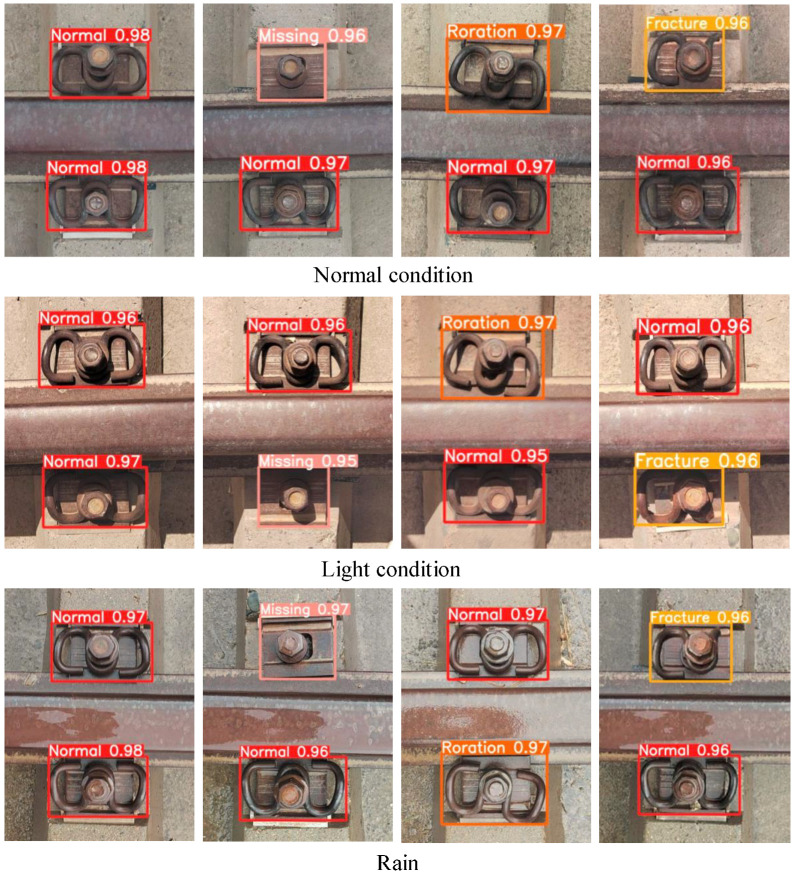
Detection effect of using the improved model in normal condition, light condition, and rain.

To verify the stability of the improved model in fastener defect detection, we obtained fastener images under normal conditions, light conditions, and rainy days in three environmental conditions and detected them using the improved model. The detection results are shown in Figure 13. The test results show that the changes in the detection accuracy of the enhanced model for fasteners under different environmental conditions could be more precise. The detection accuracy of normal fasteners can reach 98%, and the detection accuracy of defective fasteners has slightly changed, but the model’s overall performance is more stable. This experiment further verifies that the improved model has good generalization and robustness for fastener defect detection.

## 5. Conclusions

This paper addresses the lack of fastener datasets encountered in current rail fastener defect detection and the low detection efficiency of existing target detection algorithms. We built our own fastener dataset and proposed an improved YOLOv5s-based model for detecting rail fastener defects. The model introduces a CBAM module in the Neck network, which increases the model’s focus on important features. Second, to improve the feature fusion capability of the model, we used the BiFPN structure, which speeds up the fusion of semantic and positional information of the features by the model while reducing its training time. Finally, the anchor box suitable for the fastener dataset was obtained by clustering using the K-means++ algorithm, which further improves the detection accuracy of the model. After experimental validation, the mAP of the improved model reached 97.4%, which is 4.3% higher than the original model of YOLOv5s, with a detection speed of 27.3 FPS. At the same time, the improved model has good generalizability and robustness.

At present, the improved model in this paper is only suitable for defect identification of sling V fasteners and not for identification of other types of fasteners. In addition, the algorithm in this paper was not designed to lighten the model to ensure detection accuracy. Therefore, subsequent work will continue to expand the fastener dataset and increase the richness of the dataset. The model will also be studied for lightweight application and will be deployed at the edge in an attempt to realize engineering applications for rail fastener defect detection.

## Figures and Tables

**Table 1:** Comparison of different anchor box parameters.

Algorithm	Anchor Box
YOLOv5s	[10 13, 16 30, 33 23] [30 61, 62 45, 59 119] [116 90, 156 198, 373 326]
K-means	[96 151, 142 96, 175 237] [200 304, 201 150, 226 429] [284 189, 409 238, 467 516]
K-means++	[89 145, 119 174, 145 92] [154 287, 195 247, 219 152] [221 403, 314 207, 451 476]

**Table 2:** Comparison of experimental results of different attention mechanisms.

Method	P/%	R/%	mAP/%	GFLOPs
YOLOv5s	93.6	92.0	93.1	15.8
YOLOv5s_NeckSE	91.7	90.1	91.8	15.9
YOLOv5s_NeckCA	93.8	91.5	93.3	15.9
YOLOv5s_NeckCBAM	94.6	93.5	94.2	15.9

**Table 3:** Comparison of experimental results of adding CBAM at different locations.

Method	P/%	R/%	mAP/%	GFLOPs
YOLOv5s	93.6	92.7	93.1	15.8
YOLOv5s_ConvCBAM	93.9	91.3	93.4	15.9
YOLOv5s_C3CBAM	89.5	88.9	89.2	15.2
YOLOv5s_NeckCBAM	94.6	93.5	94.2	15.9

**Table 4:** Comparison of detection results of different anchors.

Algorithm	P/%	R/%	mAP/%
YOLOv5s	93.6	92.7	93.1
K-means	93.8	92.3	93.6
K-means++	94.7	94.1	94.5

**Table 5:** Comparison of results of ablation experiments.

YOLOv5s	Anchor	CBAM	BiFPN	P/%	mAP/%	GFLOPs	FPS
√	-	-	-	93.6	93.1	15.8	28.3
√	√	-	-	94.7	94.5	15.8	28.3
√	-	√	-	94.6	94.2	15.9	28.0
√	-	-	√	95.0	94.7	16.1	27.5
√	-	√	√	97.2	96.3	16.9	27.2
√	√	√	-	94.5	95.0	15.9	28.0
√	√	-	√	96.3	95.9	16.1	27.6
√	√	√	√	98.1	97.4	16.9	27.3

**Table 6:** Performance comparison of various target detection algorithms.

Algorithm	P/%	R/%	mAP/%	GFLOPs	Weight/M	FPS
Faster-R-CNN	75.1	73.4	74.6	131.5	112	2.5
SSD	82.7	78.5	79.8	90	15.0	7.1
YOLOv3	86.1	80.3	83.2	136.4	235	8.6
YOLOv6	91.8	90.5	90.6	45.1	42.5	40.2
YOLOv8	94.6	93.3	94.2	28.6	26.6	34.1
YOLOv5s	93.6	92.7	93.1	15.8	14.4	28.3
Improved YOLOv5s	98.1	96.2	97.4	16.9	15.5	27.3

## Data Availability

Not applicable.

## References

[1] Fei Y., Tu W.-J., Wei Z.-L., Ke Z.-T., Liu X.-B., Yang A.-H., Wang S.-L. (2023). Review on Development Status of Inspection Equipment for Track Maintenance. Commun. Signal. Power Supply Railw..

[2] Chellaswamy C., Krishnasamy M., Balaji L., Dhanalakshmi A., Ramesh R. (2020). Optimized Railway Track Health Monitoring System Based on Dynamic Differential Evolution Algorithm. Measurement.

[3] Zhan Z., Sun H., Yu X., Yu J., Zhao Y., Sha X., Chen Y., Huang Q., Li W.J. (2019). Wireless Rail Fastener Looseness Detection Based on MEMS Accelerometer and Vibration Entropy. IEEE Sens. J..

[4] Mao Q., Cui H., Hu Q., Ren X. (2018). A Rigorous Fastener Inspection Approach for High-Speed Railway from Structured Light Sensors. ISPRS J. Photogramm. Remote Sens..

[5] Damljanović V., Weaver R.L. (2005). Laser Vibrometry Technique for Measurement of Contained Stress in Railroad Rail. J. Sound Vib..

[6] Guerrieri M., Parla G., Celauro C. (2018). Digital Image Analysis Technique for Measuring Railway Track Defects and Ballast Gradation. Measurement.

[7] Wei X., Wei D., Suo D., Jia L., Li Y. (2020). Multi-Target Defect Identification for Railway Track Line Based on Image Processing and Improved YOLOv3 Model. IEEE Access.

[8] Viola P., Jones M. Rapid Object Detection Using a Boosted Cascade of Simple Features. Proceedings of the 2001 IEEE Computer Society Conference on Computer Vision and Pattern Recognition, CVPR 2001.

[9] Liu J., Xiong Y., Li B., Li L. (2016). Research on Automatic Inspection Algorithm for Railway Fastener Defects Based on Computer Vision. J. China Railw. Soc..

[10] Ma H., Min Y., Yin C., Cheng T., Xiao B., Yue B., Li X. (2018). A Real Time Detection Method of Track Fasteners Missing of Railway Based on Machine Vision. Int. J. Perform. Eng..

[11] Gibert X., Patel V.M., Chellappa R. Sequential Score Adaptation with Extreme Value Theory for Robust Railway Track Inspection. Proceedings of the IEEE International Conference on Computer Vision Workshops (ICCVW).

[12] Gibert X., Patel V.M., Chellappa R. (2016). Deep Multitask Learning for Railway Track Inspection. IEEE Trans. Intell. Transp. Syst..

[13] Ou Y., Luo J., Li B., He B. (2019). A Classification Model of Railway Fasteners Based on Computer Vision. Neural Comput. Appl..

[14] Dai X.-X., Ding S.-H., Yang E.-H. (2018). Development and Verification of Automatic Inspection System for High-speed Railway Fastener. J. Railw. Sci. Eng..

[15] Girshick R., Donahue J., Darrell T., Malik J. Rich Feature Hierarchies for Accurate Object Detection and Semantic Segmentation. Proceedings of the IEEE Conference on Computer Vision and Pattern Recognition (CVPR).

[16] Girshick R. Fast R-CNN. Proceedings of the 2015 IEEE International Conference on Computer Vision (ICCV).

[17] Ren S., He K., Girshick R., Sun J. (2017). Faster R-CNN: Towards Real-Time Object Detection with Region Proposal Networks. IEEE Trans. Pattern Anal. Mach. Intell..

[18] Wei X., Yang Z., Liu Y., Wei D., Jia L., Li Y. (2019). Railway Track Fastener Defect Detection Based on Image Processing and Deep Learning Techniques: A Comparative Study. Eng. Appl. Artif. Intell..

[19] Liu W., Anguelov D., Erhan D., Szegedy C., Reed S., Fu C.-Y., Berg A.C. (2016). Ssd: Single Shot Multibox Detector. Proceedings of the Computer Vision–ECCV 2016: 14th European Conference.

[20] Redmon J., Divvala S., Girshick R., Farhadi A. You Only Look Once: Unified, Real-Time Object Detection. Proceedings of the IEEE Conference on Computer Vision and Pattern Recognition (CVPR).

[21] Redmon J., Farhadi A. YOLO9000: Better, Faster, Stronger. Proceedings of the IEEE Conference on Computer Vision and Pattern Recognition (CVPR).

[22] Redmon J., Farhadi A. (2018). Yolov3: An Incremental Improvement. arXiv.

[23] Bochkovskiy A., Wang C.-Y., Liao H.-Y.M. (2020). Yolov4: Optimal Speed and Accuracy of Object Detection. arXiv.

[24] Qi H., Xu T., Wang G., Cheng Y., Chen C. (2020). MYOLOv3-Tiny: A New Convolutional Neural Network Architecture for Real-Time Detection of Track Fasteners. Comput. Ind..

[25] Guo F., Qian Y., Shi Y. (2021). Real-Time Railroad Track Components Inspection Based on the Improved YOLOv4 Framework. Autom. Constr..

[26] Jocher G. (2020). YOLOv5 by Ultralytics. https://github.com/ultralytics/yolov5.

[27] Chollet F. Xception: Deep Learning with Depthwise Separable Convolutions. Proceedings of the IEEE Conference on Computer Vision and Pattern Recognition.

[28] Lin T.-Y., Dollar P., Girshick R., He K., Hariharan B., Belongie S. Feature Pyramid Networks for Object Detection. Proceedings of the IEEE Conference on Computer Vision and Pattern Recognition.

[29] Hu J., Shen L., Sun G. Squeeze-and-excitation networks. Proceedings of the IEEE Conference on Computer Vision and Pattern Recognition.

[30] Hou Q., Zhou D., Feng J. Coordinate attention for efficient mobile network design. Proceedings of the IEEE/CVF Conference on Computer Vision and Pattern Recognition.

[31] Woo S., Park J., Lee J.-Y., Kweon I.S. CBAM: Convolutional Block Attention Module. Proceedings of the European Conference on Computer Vision (ECCV).

[32] Liu S., Qi L., Qin H., Shi J., Jia J. (2018). Path Aggregation Network for Instance Segmentation. arXiv.

[33] Lin T.-Y., Maire M., Belongie S., Hays J., Perona P., Ramanan D., Dollár P., Zitnick C.L. (2014). Microsoft COCO: Common Objects in Context.

[34] Kisantal M., Wojna Z., Murawski J., Naruniec J., Cho K. (2019). Augmentation for Small Object Detection. arXiv.

[35] GitHub: LabelImg: A Graphical Image Annotation Tool—Google. https://github.com/tzutalin/labelImg.

[36] Everingham M., Van Gool L., KI W.C., Winn J., Zisserman A. (2010). The PASCAL Visual Object Classes (VOC) Challenge. Int. J. Comput. Vis..

[37] Padilla R., Netto S.L., Da Silva E.A. A Survey on Performance Metrics for Object-Detection Algorithms. Proceedings of the 2020 International Conference on Systems, Signals and Image Processing (IWSSIP).

[38] Shah T. (2018). Measuring Object Detection Models—MAP—What Is Mean Average Precision. Tarang Shah-Blog.

[39] Chu Y.L., Hong L.J., Kai H.W. YOLOv6: A Single-Stage Object Detection Framework for Industrial Applications. Proceedings of the 2022 IEEE Conference on Computer Vision and Pattern Recognition (CVPR).

[40] Jocher G., Chaurasia A., Qiu J. (2023). YOLO by Ultralytics. https://github.com/ultralytics/ultralytics.

